# Dr. Wm. G. Maguire

**Published:** 1913-02

**Authors:** 


Dr. Wm. G. Maguire (years of practice) died in Tuscola, Ill., November 29, 1912, aged 99. He was the father of twenty-three children.

